# Monitoring AKT activity and targeting in live tissue and disease contexts using a real-time Akt-FRET biosensor mouse

**DOI:** 10.1126/sciadv.adf9063

**Published:** 2023-04-26

**Authors:** James R. W. Conway, Sean C. Warren, Young-Kyung Lee, Andrew T. McCulloch, Astrid Magenau, Victoria Lee, Xanthe L. Metcalf, Janett Stoehr, Katharina Haigh, Lea Abdulkhalek, Cristian S. Guaman, Daniel A. Reed, Kendelle J. Murphy, Brooke A. Pereira, Pauline Mélénec, Cecilia Chambers, Sharissa L. Latham, Helen Lenthall, Elissa K. Deenick, Yuanqing Ma, Tri Phan, Elgene Lim, Anthony M. Joshua, Stacey Walters, Shane T. Grey, Yan-Chuan Shi, Lei Zhang, Herbert Herzog, David R. Croucher, Andy Philp, Colinda L.G.J. Scheele, David Herrmann, Owen J. Sansom, Jennifer P. Morton, Antonella Papa, Jody J. Haigh, Max Nobis, Paul Timpson

**Affiliations:** ^1^ Garvan Institute of Medical Research & The Kinghorn Cancer Centre, Sydney, NSW 2010, Australia.; ^2^ St Vincent’s Clinical School, Faculty of Medicine, UNSW Sydney, Sydney, NSW 2010, Australia.; ^3^ Turku Bioscience Centre, University of Turku and Åbo Akademi University, FI-20520 Turku, Finland.; ^4^ Cancer Ecosystems Program, Garvan Institute of Medical Research, Sydney, NSW 2010, Australia.; ^5^ School of Clinical Medicine, UNSW Sydney, Randwick Clinical Campus, Sydney, NSW, Australia.; ^6^ Department of Pharmacology and Therapeutics, Rady Faculty of Health Sciences, University of Manitoba, Winnipeg, Manitoba, Canada.; ^7^ CancerCare Manitoba Research Institute, Winnipeg, Manitoba, Canada.; ^8^ School of Clinical Medicine, Randwick Clinical Campus, UNSW Sydney, Centre for Healthy Ageing, Centenary Institute, Missenden Road, Sydney, NSW 2050, Australia.; ^9^ Charles Perkins Centre, Faculty of Medicine and Health, University of Sydney, Sydney, NSW 2006, Australia.; ^10^ Laboratory for Intravital Imaging and Dynamics of Tumor Progression, VIB Center for Cancer Biology, KU Leuven, 3000 Leuven, Belgium.; ^11^ Department of Oncology, KU Leuven, 3000 Leuven, Belgium.; ^12^ Cancer Research UK Beatson Institute, Glasgow G611BD, UK.; ^13^ School of Cancer Sciences, Wolfson Wohl Cancer Research Centre, Institute of Cancer Sciences, University of Glasgow, Glasgow G611QH, UK.; ^14^ Monash Biomedicine Discovery Institute and Department of Biochemistry and Molecular Biology, Monash University, Melbourne, VIC 3800, Australia.; ^15^ Intravital Imaging Expertise Center, VIB Center for Cancer Biology, KU Leuven, 3000 Leuven, Belgium.

## Abstract

Aberrant AKT activation occurs in a number of cancers, metabolic syndrome, and immune disorders, making it an important target for the treatment of many diseases. To monitor spatial and temporal AKT activity in a live setting, we generated an Akt-FRET biosensor mouse that allows longitudinal assessment of AKT activity using intravital imaging in conjunction with image stabilization and optical window technology. We demonstrate the sensitivity of the Akt-FRET biosensor mouse using various cancer models and verify its suitability to monitor response to drug targeting in spheroid and organotypic models. We also show that the dynamics of AKT activation can be monitored in real time in diverse tissues, including in individual islets of the pancreas, in the brown and white adipose tissue, and in the skeletal muscle. Thus, the Akt-FRET biosensor mouse provides an important tool to study AKT dynamics in live tissue contexts and has broad preclinical applications.

## Teaser

The Akt-FRET biosensor mouse reveals live spatiotemporal regulation in cancer, metabolism and whole-body molecular targeting.

## INTRODUCTION

AKT is a central signaling node controlling a plethora of cellular processes, such as cell survival, proliferation, migration, and metabolism (*1*–*3*). To achieve a robust quantification of AKT activity in live tissue, we used an Eevee-Akt-FRET (Förster resonance energy transfer) biosensor (*4*, *5*) to generate the first inducible Akt-FRET biosensor mouse. Using our Akt-FRET biosensor mouse, we performed spatiotemporal analysis of AKT activity in a variety of tissue contexts where rapid time-resolved longitudinal analysis of AKT signaling has been historically difficult to achieve by conventional biochemical methods in live organ systems in vivo.

AKT [protein kinase B (PKB)] is related to PKA and PKC, as demonstrated by the comparison to the viral homolog v-AKT (*6*–*8*). AKT further contains an N-terminal pleckstrin homology (PH) domain, which allows for binding to the second messenger phosphatidylinositol-3,4,5-trisphosphate (PIP_3_) at cell membranes. Activation of the upstream phosphoinositide 3-kinase (PI3K) leads to the phosphorylation of phosphatidylinositol-4,5-bisphosphate (PIP_2_) to generate the second messenger PIP_3_, resulting in the recruitment of AKT to the membrane and in its full activation (*9*, *10*). This activity, in turn, is regulated by the action of cellular phosphatases, such as the tumor suppressor phosphatase and tensin homolog detected on chromosome 10 (PTEN) (*11*).

The regulation of cancer and metabolism by AKT has been extensively reviewed and has illustrated the vast network through which AKT exerts control in malignant transformation and metabolism (*12*). AKT activity is induced by G protein–coupled receptors (GPCRs), receptor tyrosine kinases (RTKs), integrins, and cytokine receptors and is a vital signaling node in a number of signal transduction pathways involved in disease etiology (*13*). Aberrant AKT activity can be achieved through multiple genetic alterations including (i) gain-of-function missense mutations and amplifications of the AKT gene, (ii) activating mutations in *PIK3CA*, encoding for the p110α catalytic subunit of PI3K, (iii) activation and amplification of specific PI3K-activating RTKs, and (iv) loss-of-function mutations or deletions in PTEN, which constitutes the second most lost or mutated tumor suppressor gene after *TP53* (*14*, *15*).

We have previously demonstrated that AKT activity in pancreatic cancer is associated with loss of PTEN (*16*) or hypoxic regions in primary tumors (*3*–*5*). In this study, we have generated the first inducible Akt-FRET biosensor mouse to readily examine live AKT signaling in genetically engineered mouse models (GEMMs) of disease. First, we used our novel Akt-FRET biosensor mouse to measure and map AKT activity in the native tumor context of PTEN-deficient pancreatic ductal adenocarcinoma (PDAC) tumors. Elevated AKT activity has also been described in prostate and mammary carcinomas, driven by PTEN loss (*17*). Here, PTEN knockout mice (*Pten^−/+^*) or knock-in mice expressing the loss-of-function and cancer-associated PTEN^G129E^ point mutation (*Pten^G129E/+^*) (*17*) were crossed to our Akt-FRET biosensor mice to map the activation status of AKT in these established long-term tumor models. Moreover, we used spheroids derived from the prostate tumor Akt biosensor model to prescreen clinically relevant combination treatments in vitro. In a PTEN loss or mutated breast cancer setting, we also use the Akt-FRET biosensor mouse to assess in vivo targeting efficacy of the AKT-PI3K signaling axis in a live disease context. This was coupled with high-throughput assessment of primary Akt-FRET cancer cells in three-dimensional (3D) organotypic invasion assays to validate the anti-invasive capacity of AKT-PI3K pathway inhibition. Furthermore, using optical window imaging (*18*–*21*) in breast cancer GEMMs, we mapped drug targeting in an AKT hyperactivated setting, demonstrating the utility of the Akt-FRET biosensor mouse in preclinical imaging of targeted therapies in a longitudinal manner (*22*–*24*).

In addition to assessing AKT signaling in cancer, we also assess how the biosensor mouse can be used to monitor AKT activity in other disease settings. For example, genetic deletion of AKT has previously been shown to lead to severe insulin resistance (*25*) and the development of type 2 diabetes (*26*), clearly demonstrating the importance of AKT for insulin and glucose response in vivo. Here, AKT activity directly regulates downstream effectors including members of the forkhead box O (FOXO) transcription factors, mechanistic target of rapamycin complex 1 (mTORC1), and glycogen synthase kinase 3 (GSK3) (*27*). Glucose transport into cells is regulated, in turn, by glucose transporter family (GLUT) proteins. In particular, GLUT2 is expressed in pancreatic islet beta cells and, upon high glucose concentration in the small intestine, is activated to translocate glucose (*28*). Another transporter GLUT4 is expressed in insulin-responsive cells such as muscle and adipose tissue (*29*). However, monitoring how these processes are regulated by AKT signaling dynamics in a live tissue setting in vivo has not been demonstrated before and is robustly quantified here using the Akt-FRET biosensor mouse in a spatiotemporal manner. In particular, we show live metabolic monitoring of AKT-controlled glucose and insulin responses in individual islets using abdominal imaging windows (AIWs) to image deep within the abdominal cavity and facilitate live pancreatic tissue imaging, which cannot be accurately achieved from in vitro assessment. Moreover, we assess AKT response kinetics in brown adipose tissue (BAT) and white adipose tissue (WAT) in response to glucose and insulin while also tracking AKT activity in muscle in this context. Collectively, we demonstrate the utility of the Akt-FRET biosensor mouse in monitoring AKT activity in native tissue settings and illustrate the broad spectrum of applications this new Akt-FRET biosensor mouse provides for accurate tracking of AKT signaling in any organ or tissue of interest.

## RESULTS

### Generation and characterization of the Akt-FRET biosensor mouse in a variety of tissues

**Fig. 1.**
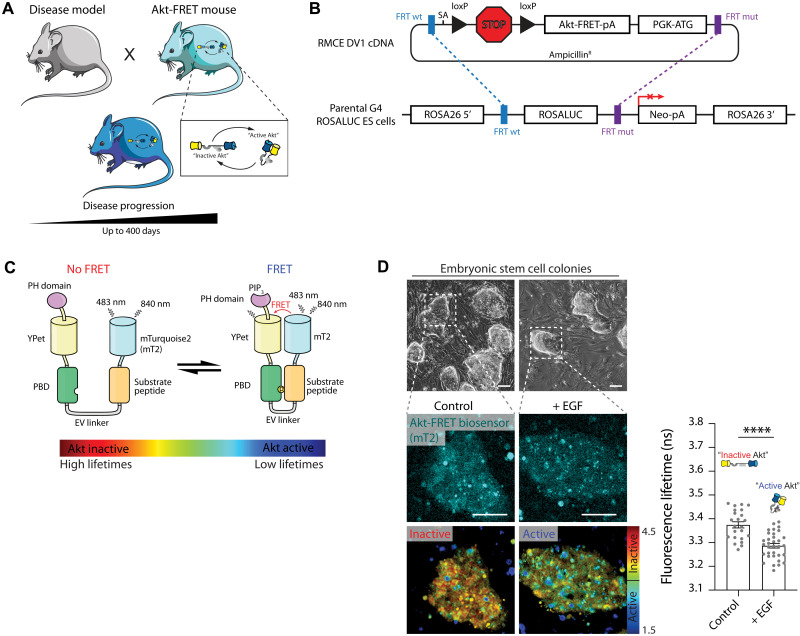
Generation of the Akt-FRET biosensor mouse. (**A**) Schematic of the Akt-FRET biosensor mouse crossed to various tissue-specific Cre-driver lines and disease models with latencies exceeding up to 400 days. The Eevee-Akt-FRET biosensor that gets phosphorylated by active AKT in the cells can be used in conjunction with optical imaging windows to monitor AKT activity in a spatiotemporally resolved manner in vivo. (**B**) Targeting of the Akt-FRET biosensor to the ROSA26 locus via recombinase-mediated cassette exchange (RMCE) via heterospecific FRT sites. (**C**) Schematic of the Eevee-Akt-FRET biosensor comprising mTurquoise2 (mT2) and YPet in its inactive (no FRET) and active (FRET) conformation upon phosphorylation of the substrate peptide by active AKT in cells. (**D**) Phase-contrast images of embryonic stem cell (ESC) colonies isolated from Akt-FRET biosensor mice cocultured with mouse embryonic fibroblasts (MEFs), before and after EGF stimulation, with corresponding intensity images (mTurquoise2, cyan) and FLIM images. *n* = 23 to 33 colonies per condition. Results are means ± SEM. *P* value was determined using Welch’s *t* test, and significance is compared to untreated control colonies. Scale bars, 50 μm. ****P < 0.0001.

To spatiotemporally monitor AKT signaling in organ-specific homeostasis and disease contexts, we generated an Akt-FRET biosensor mouse using the mTurquoise2-YPet–modified Eevee-Akt-FRET biosensor described and characterized previously (*4*, *5*). The construct was flanked by a loxP-stop-loxP domain to allow for conditional expression of the biosensor in selected tissues of interest and was targeted to the *Rosa26* locus using a recombinase-mediated cassette exchange (RMCE)–compatible flip/flippase recognition target (FRT) system (Fig. 1, A and B) (*30*, *31*). When AKT is activated in cells, it phosphorylates the AKT consensus substrate peptide within the biosensor, leading to a conformational change after binding to the phosphopeptide-binding domain (PBD) of FHA1. The resulting juxtaposition of the donor mTurquoise2 to the acceptor YPet leads to an increase in FRET efficiency, which can be measured by fluorescence lifetime imaging microscopy (FLIM) (Fig. 1C) (*2*, *22*, *32*). To initially test the biosensor in embryonic stem cell (ESC) colonies, isolated ESCs were treated in vitro with epidermal growth factor (EGF; 50 ng/ml), showing a significant decrease in fluorescence lifetime as expected upon activation of AKT [Fig. 1D; lifetime colormaps represent inactive AKT (low-FRET) by red/yellow colors, while areas of active AKT (high-FRET) are represented as green/blue colors and black depicting areas of no signal]. Here, using FLIM imaging allows us to quantitatively assess the activity of AKT in complex 3D environments, which would be difficult to achieve using other methods such as ratiometric FRET. We next crossed the Akt-FRET mouse (AKT-FRET “OFF” mice) to cytomegalovirus (CMV)–Cre recombinase–expressing mice to enable ubiquitous expression of the Akt-FRET biosensor (referred to herein as Akt-FRET “ON” mice). Homozygous offspring of both ubiquitous expression and loxP-stop-loxP (LSL) retaining mice were healthy and fertile, showed no abnormal defects, and exhibited the expected Mendelian ratio of hereditary transmission. Mice generated using the biosensor were assessed for expression of the biosensor, which was confirmed by orthogonal analyses using Western blotting and anti–green fluorescent protein (GFP) immunohistochemistry (IHC) in various organs of the Akt-FRET ON mouse (fig. S1, A to G, and movie S1). Endogenous AKT levels and activity were found to be unaltered by the low-level presence of the Akt biosensor in multiple tissue settings.

### Suborgan-specific AKT activity revealed in the pancreas and pancreatic cancer

To illustrate the inducible lox-stop-lox tissue-specific capacity of the biosensor mouse, expression in suborgan compartments of neuroendocrine organs was induced via a neuropeptide-Y lineage–specific Cre recombinase model crossed to the Akt-FRET biosensor mouse (fig. S1H). This allowed us to quantify AKT activity specifically in neurons of the brain (fig. S1I).

**Fig. 2.**
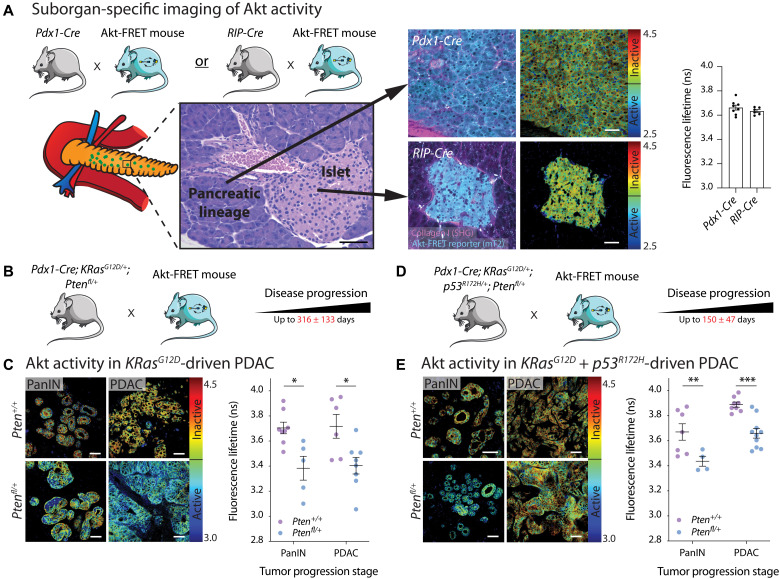
Suborgan-specific mapping of AKT activity in the pancreas and pancreatic islets as well as PDAC in the context of PTEN loss. (**A**) Schematic of suborgan-specific expression of the Akt-FRET biosensor in cells of pancreatic lineage (*Pdx1-Cre*) or islets (*RIP-Cre*), showing Akt-FRET biosensor (mTurquoise2, cyan) and second-harmonic generation (SHG; magenta) with corresponding representative intensity-merged maps of mTurquoise2 fluorescence lifetime. *n* = 2 to 4 mice with six to eight images per islets and 136 cells in total quantified. Results are means ± SEM. (**B** to **E**) Increasing AKT activity in *KRas^G12D^*-driven (B and C) and *KRas^G12D^* + *p53^R172H^*–driven (D and E) PanINs and PDAC upon heterozygous loss of *Pten* as imaged ex vivo. *n* = 4 to 10 mice per condition and 1460 cells in total quantified. Results are means ± SEM. *P* values were determined using an ordinary two-way ANOVA with Šídák’s correction for multiple comparisons, and significance is compared to *Pten* “+/+” mice. Scale bars, 50 μm. ns (not significant), *P* > 0.05, *P < 0.05, **P < 0.01, and ***P < 0.001.

Having confirmed that the Akt-FRET biosensor does not perturb endogenous signaling in the pancreas (fig. S1, A and B), we next sought to explore the role of AKT in this organ by compartment-specific expression of the Akt-FRET biosensor in all cells of the pancreas or solely within pancreatic islets. The Akt-FRET biosensor mouse was therefore crossed to pancreas lineage–specific Cre driver line Pdx1-Cre or RIP-Cre–specific expression in the beta cells of pancreatic islets, with AKT activity quantified in both models (Fig. 2A, expression confirmed by IHC; fig. S2A). To test the response to glucose stimulation, isolated pancreatic islets were initially challenged with 20 mM glucose ex vivo and AKT activity was measured. This showed a strong activation of AKT in the islets (fig. S2B). PDAC has been strongly associated with increased AKT activity, with over 60% of PDAC samples showing elevated AKT activity and 10 to 20% of PDAC cases presenting with amplification of the AKT2 oncogene (*3*).

We have previously demonstrated that the PI3K/AKT pathway is involved in driving PDAC tumorigenesis in RAS-mutated tumors by antagonizing KRas-induced senescence, and that elevated AKT signaling is observed upon the loss of the tumor suppressor PTEN (*16*, *33*). To monitor AKT activity in the context of pancreatic cancer progression, the biosensor mouse was crossed to the *KRas^G12D/+^;Pdx1-Cre* (KC) or *KRas^G12D/+^;p53^R172H/+^;Pdx1-Cre* (KPC) models of PDAC, respectively. The KC and KPC models faithfully recapitulate the progression of human PDAC in mice (*34*, *35*). Mice were allowed to develop pancreatic cancer, which occurred with latencies of 316 ± 133 days (KC) and 150 ± 47 days (KPC) (Fig. 2, B to E). Heterozygous loss of *Pten* in KC and KPC mice led to a marked up-regulation of AKT activity in early stages of disease progression, as observed here in pancreatic intraepithelial neoplasms [PanINs; Fig. 2C (left) for KC and Fig. 2E (left) for KPC]. This enhanced activity was also maintained at late stages of the disease, as assessed in PDAC tissue by multiphoton-based FLIM-FRET imaging [Fig. 2C (right) for KC and Fig. 2E (right) for KPC]. Having demonstrated the utility of the Akt-FRET biosensor mouse in assessing aberrant AKT activity when a tumor suppressor is lost in pancreatic cancer, we went on to additionally show the effect of an activating mutation of PI3K upstream of AKT signaling in the context of immunodeficiency. The delta variant of p110, p100δ (*PIK3CD*), which is restricted to the leukocyte lineage, governs several aspects of immunity, and gain-of-function mutations of *PIK3CD* at residue E1020K have been described previously to cause activated PI3K-δ syndrome (APDS) characterized with recurrent respiratory infections, viremia, lymphadenopathy, and nodular lymphoid hyperplasia. Hyperactivation of AKT was observed in the spleens of mice imaged live in vivo here in Akt-FRET biosensor mice crossed to a *Pi3kcd^E1020K^* gain-of-function mouse model (fig. S2C) (*36*).

### Nonsteroidal antiandrogen therapy enzalutamide increases AKT activity in Pten^G129E^ mutant prostate cancer and can be counteracted by BKM120 inhibition

**Fig. 3.**
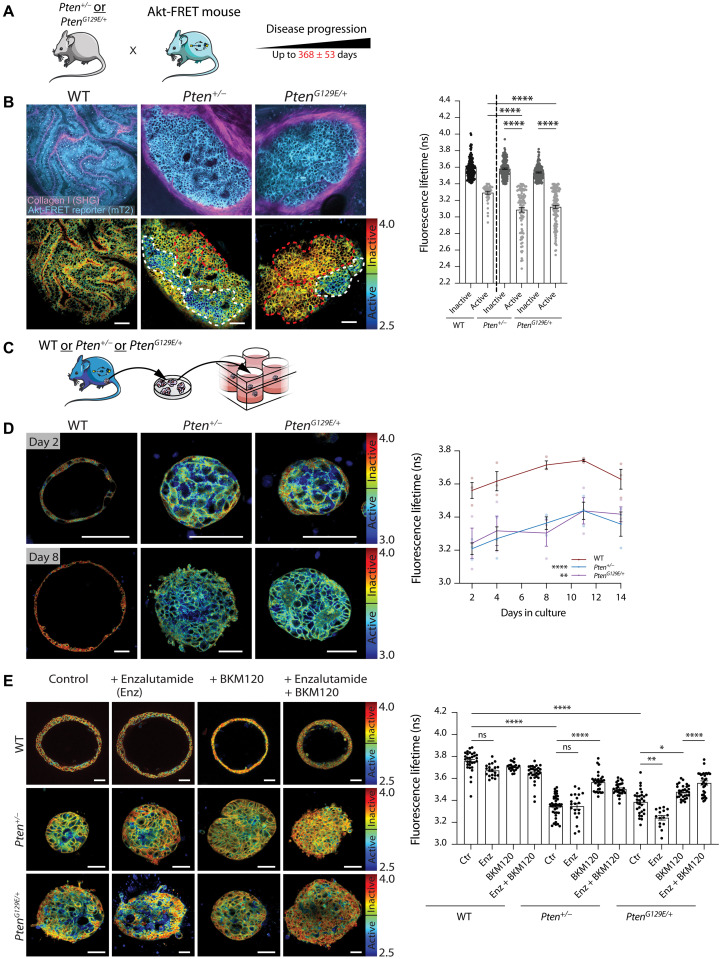
Standard of care nonsteroidal antiandrogen therapy enhances AKT signaling in *Pten^G129E^* mutant prostate cancer and can be combatted by BKM120 inhibition. (**A**) Schematic of crossing the Akt-FRET biosensor mouse to whole-body *Pten^+/−^* or *Pten^G129E/+^* mutant mice and indicated latency to disease progression of 368 ± 53 days. (**B**) Increasing AKT activity in locally restricted zones of prostate cancers following heterozygous PTEN loss (*Pten^+/−^*) and mutant PTEN expression (*Pten^G129E/+^*). *n* = 6 mice per genotype, 780 cells in total analyzed (white dotted lines: AKT active cells, red dotted lines: AKT inactive cells, cutoff value for activity at 3.4 ns). Results are means ± SEM. *P* values were determined using Brown-Forsythe and Welch ANOVA tests for multiple comparisons, and significance is compared to WT prostate active population and *Pten^+/−^* and *Pten^G129E/+^* inactive populations, respectively. (**C**) Isolation schematic of prostate cancer spheroids and culture in Matrigel. (**D**) Prostate spheroids cultured for up to 14 days with representative images on days 2 and 8 showing maintained increased AKT activity in *Pten^+/−^* and *Pten^G129E/+^* spheroids. *n* = 3 spheroids per condition per time point, 1274 cells in total. Results are means ± SEM. *P* values were determined using repeated-measures one-way ANOVA with a Geisser-Greenhouse and Dunnett correction for multiple comparisons, and significance is compared to WT spheroids. (**E**) Treatment of prostate cancer spheroids with enzalutamide (Enz) shows enhanced AKT activation in *Pten^G129E/+^* spheroids. AKT activity is effectively inhibited with BKM120 alone and in combination with enzalutamide. *n* = 1 to 3 spheroids per condition, 325 cells in total analyzed. Results are means ± SEM. *P* values were determined using Brown-Forsythe and Welch ANOVA with Dunnett correction for multiple comparisons, and significance is compared to the respective groups indicated in the graph. Scale bars, 50 μm. ns, *P* > 0.05, *P < 0.05, **P < 0.01, and ****P < 0.0001.

The PI3K/AKT signaling pathway is altered in approximately 40% of early prostate neoplasms and 70 to 100% in advanced prostate cancer (*37*, *38*). Loss of the tumor suppressor PTEN in particular has been shown in 30% of primary tumors and in up to 60% of castration-resistant prostate cancer (*39*). Heterozygous loss of *Pten* has been shown to lead to prostate cancer development in a variety of mouse models of cancer (*40*). To monitor AKT activation in a PTEN loss–driven prostate cancer model, the Akt-FRET biosensor mice were crossed with either the *Pten^−/+^* model or the mutant *Pten^G129E/+^* knock-in mouse line expressing a lipid phosphatase dead PTEN. For both models, PTEN inactivation initiated a range of neoplastic transformation of epithelial cells and caused lymphoproliferative disorders, as previously reported (*17*). Malignancies that form in these models include lymphoadenophathy and adrenal tumors, in which AKT activity at a single-cell and subpopulation level was readily measured (fig. S3, A and B). Older cohorts of mice, with an average of 368 ± 53 days of age, also displayed neoplastic lesions of the prostate, which showed spatially restricted up-regulated AKT activity (Fig. 3, A and B; white dotted lines: AKT active cells, red dotted lines: AKT inactive cells). The use of the Akt-FRET biosensor mouse here allowed for the quantitative mapping of the spatial heterogeneity of AKT signaling. To test how standard-of-care treatments based on nonsteroidal antiandrogen therapy affected AKT activation, we isolated primary prostate spheroids from developed prostate tumors and cultured them as described previously (*41*). These prostate cancer spheroids were subsequently used as a drug treatment screening platform to screen previously unidentified combinatorial treatments in the context of specific genetic models of PTEN loss or mutation. First, we found that isolated spheroids from *Pten^−/+^* and mutant *Pten^G129E/+^* mice (Fig. 3C) retained higher AKT signaling during 14 days of culture, compared to wild-type cells (Fig. 3D).

Second, we used targeted treatments directed at the androgen receptor (AR), which is intrinsically linked to PI3K pathway signaling in prostate cancer and whereby AR inhibition leads to an up-regulation of AKT activity via suppression of the AKT phosphatase PHLPP (*37*). Wild-type (WT) spheroids, which alone developed a lumen, did not show any enhancement in AKT signaling as observed in vivo (Fig. 3, B and E). Conversely, AKT activity was up-regulated in *Pten^−/+^* spheroids to the same extent before and after treatment with the nonsteroidal antiandrogen enzalutamide (Fig. 3E). In mutant PTEN (*Pten^G129E/+^*) spheroids, the treatment with enzalutamide resulted in further activation of AKT compared to untreated controls. To counteract this activation of AKT in a PTEN loss or mutant background, treatment with a pan-PI3K inhibitor, BKM120 (*42*), resulted in effective inactivation of AKT as monotherapy, as well as when given in combination with enzalutamide (Fig. 3E). This demonstrates the additional utility of the Akt-FRET biosensor mouse in monitoring and screening the dynamics of the AKT-PI3K signaling axis in various genetic backgrounds that mimic the long-term disease setting, while also allowing for both in vivo and ex vivo spheroid assays to be performed for disease-specific molecular drug combination assessments using live imaging.

### PI3K inhibitor BKM120 effectively inhibits elevated AKT activity in mammary carcinoma and decreases cancer cell invasion

**Fig. 4.**
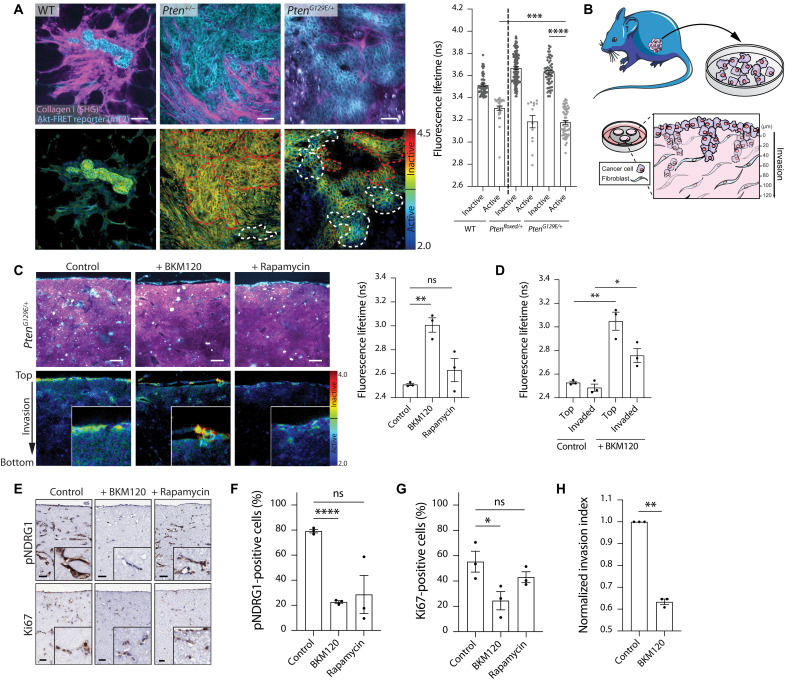
AKT activity is elevated in mutant *Pten^G129E/+^*–driven mammary carcinomas and effectively inhibited by BKM120 treatment in 3D invasion assays. (**A**) AKT activity in cell populations of *Pten^+/−^*- and mutant *Pten^G129E/+^*–driven mammary carcinomas compared to the WT mammary gland. *n* = 3 to 4 mice per genotype, 335 cells quantified (white dotted lines: AKT active cells, red dotted lines: AKT inactive cells, cutoff value for activity at 3.4 ns). Results are means ± SEM. *P* values were determined using Brown-Forsythe and Welch ANOVA tests for multiple comparisons, and significance is compared to WT mammary gland active population and the *Pten^G129E/+^* inactive population. Scale bars, 50 μm. (**B**) Schematic of cell line isolation from primary mammary tumors and setup of 3D organotypic invasion assays. (**C**) Treatment of a mutant *Pten^G129E/+^* cell line invading on organotypic matrices with BKM120 and rapamycin with corresponding quantifications of AKT activity. *n* = 3, 333 cells quantified. Results are means ± SEM. *P* values were determined using ordinary one-way ANOVA. Scale bars, 50 μm. (**D**) Breakdown of AKT activity in cell populations on top of the matrices or invaded ± treatment with BKM120 [data from (C)]. Results are means ± SEM. *P* values were determined using ordinary one-way ANOVA, and significance is compared to untreated controls. (**E**) Representative images of phospho-NDRG1 (pNDRG1) and Ki67 staining performed on a *Pten^G129E/+^* cell line invading on organotypic matrices ± BKM120; rapamycin quantified in (**F**) and (**G**). *n* = 3 per cell line. (**H**) Normalized invasion index of mutant *Pten^G129E^* cells invading on organotypic matrices ± BKM120. *n* = 3 per treatment condition. Results are means ± SEM. *P* values were determined using Welch’s *t* test, and significance is compared to untreated controls. Scale bars, 50 μm. ns, *P* > 0.05, *P < 0.05, **P < 0.01, ***P < 0.01, and ****P < 0.0001.

Loss of PTEN by missense mutations, deletion, or silencing has been additionally reported in many breast cancer subtypes (*43*). To monitor the aberrant AKT activity in this setting, Akt-FRET biosensor mice were crossed with the *Pten^−/+^* or mutant *Pten^G129E/+^* models, which further developed mammary tumors by 377 ± 137 days. Mutant *Pten^G129E/+^* tumors in particular demonstrated up-regulation in AKT activity in a subset of cells at the border of tumors (Fig. 4A, white dotted lines: AKT active cells, red dotted lines: AKT inactive cells). Here, again, the use of the novel Akt-FRET biosensor mouse allowed us to map spatially distinct AKT signaling heterogeneity in these *Pten^−/+^*- or mutant *Pten^G129E/+^*–driven breast cancer tumors. Primary tumor–derived cell lines were subsequently isolated (Fig. 4B) and further tested on 3D organotypic invasion assays (*44*). Here, we could readily assess the invasive capacity of these cells to invade into the matrices for 14 days and observed that AKT was up-regulated during invasion (Fig. 4C) with no correlation of AKT activity and depth of invasion of the cells observed (fig. S4A). To examine the effect of inhibiting the AKT-PI3K pathway on breast cancer invasion in this 3D context, mutant *Pten^G129E/+^* cells were allowed to invade and were treated with either the pan-PI3K inhibitor BKM120, which acts upstream of AKT, or the downstream inhibitor rapamycin, targeting mTORC1 (fig. S4B). We found that a marked reduction in AKT activity occurred in BKM120-treated cells, but not for rapamycin-treated matrices (Fig. 4C) (*5*). AKT activity was further quantified in cells both on top of the matrix and in those that had invaded into the matrices, before and after BKM120 inhibition. This showed that BKM120 effectively inhibited both noninvaded and invaded cells, demonstrating the efficacy of this inhibitor at targeting aberrant AKT activity in the context of both nonmotile and migratory cancer cell movement (Fig. 4, D and H). In all experimental conditions, we also monitored the phosphorylation status of NDRG1 as an orthogonal readout and found a significant reduction in the activation of this parallel signaling pathway in BKM120-treated conditions compared to the untreated control. In the rapamycin-treated matrices, a broad distribution of pNDRG1 staining was observed, which resulted in a nonsignificant difference in overall staining when compared to the untreated controls (Fig. 4, E and F). In parallel, pS6 staining, acting downstream of the mTORC1 complex, showed an expected reduction in cells in the rapamycin-treated matrices, but not BKM120 treatment conditions (fig. S4, C and D). Proliferation was also measured by Ki67 staining, which was only significantly reduced in BKM120-treated matrices (Fig. 4G). Treatment of the 3D organotypic matrices with BKM120 further decreased the overall invasion in these cells (Fig. 4H). As BKM120 inhibited AKT activity not only in the noninvasive (top) cells, but also in the cells that had invaded into the 3D collagen matrix, while additionally reducing proliferation of these cells, we subsequently went on to assess the inhibition kinetics of BKM120 in vivo.

**Fig. 5.**
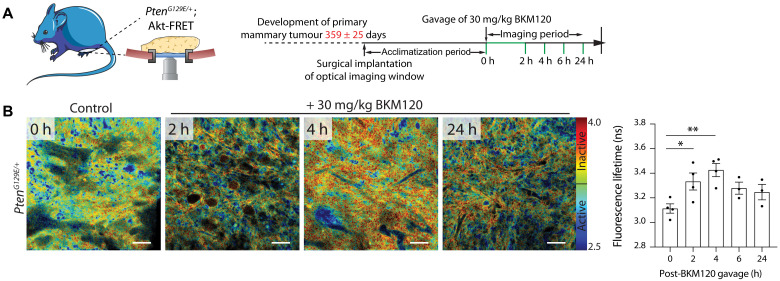
Monitoring of effective inhibition of elevated AKT activity in mutant *Pten^G129E/+^*–driven mammary carcinomas by BKM120 treatment in vivo. (**A**) Schematic of implantation of optical imaging windows over developed primary mutant *Pten^G129E/+^*–driven mammary carcinomas, treatment with BKM120, and imaging time intervals after treatment. (**B**) Time course of BKM120 treatment (30 mg/kg) imaged through optical imaging windows in primary mammary carcinomas, quantifying AKT activity at 0, 2, 4, 6, and 24 hours after treatment. *n* = 3 to 4 mice per time point, 609 cells in total quantified. Results are means ± SEM. *P* values were determined using ordinary one-way ANOVA with Dunnett correction for multiple comparisons, and significance is compared to 0-hour time point. Scale bars, 50 μm. ns, *P* > 0.05, *P < 0.05, and **P < 0.01.

To measure the effects of BKM120 in an in vivo setting, *Pten^G129E/+^*;Akt-FRET mice were allowed to develop primary mammary carcinomas for up to 359 ± 25 days and engrafted with a mammary imaging window (MIW) over the primary tumor (Fig. 5A). Inherent physiological motion experienced during intravital imaging such as the heartbeat, respiration, and blood flow during optical imaging window imaging can often interfere with single cell–based FLIM-FRET measurement of AKT activity in live organ systems (*23*, *45*). To overcome this limitation, we used our in-house image stabilization software Galene (*46*) (movie S2). We successfully corrected for these movement artifacts using Galene, allowing for reliable assessment of AKT activity in primary tumors. Mice with engrafted optical imaging windows placed over developed primary mammary tumors were treated with BKM120 (30 mg/kg by oral gavage), and AKT activity was measured longitudinally at 2, 4, 6, and 24 hours after treatment. This revealed effective inhibition of AKT activity after 2 hours, with maximal inhibition kinetics at 4 hours, which reverted to baseline AKT activity at 6 to 24 hours after gavage (Fig. 5B). Collectively, these data (Fig. 4, A to C and J) demonstrated the effective molecular guided targeting and inhibition of AKT activity and invasive capacity of mutant *Pten*^*G129E/*+^ cancer cells by treatment with the pan-PI3K inhibitor BKM120 in a time-resolved manner. Uncovering these spatially and temporally distinct AKT activation dynamics can now help guide how often treatment is required to effectively inhibit AKT in live tumor settings and allow on-target efficacy studies to be carried out in the tissue of interest. Aided by image-guided validation of the inhibition in both 3D organotypic assays in vitro and intravital imaging of the primary tumor in vivo, several additional upstream or downstream targets can be assessed reliably in this manner in the future using the Akt-FRET biosensor mouse.

### Longitudinal, spatiotemporal imaging of metabolically regulated AKT activation monitored in vivo using optical AIWs in live tissues

**Fig. 6.**
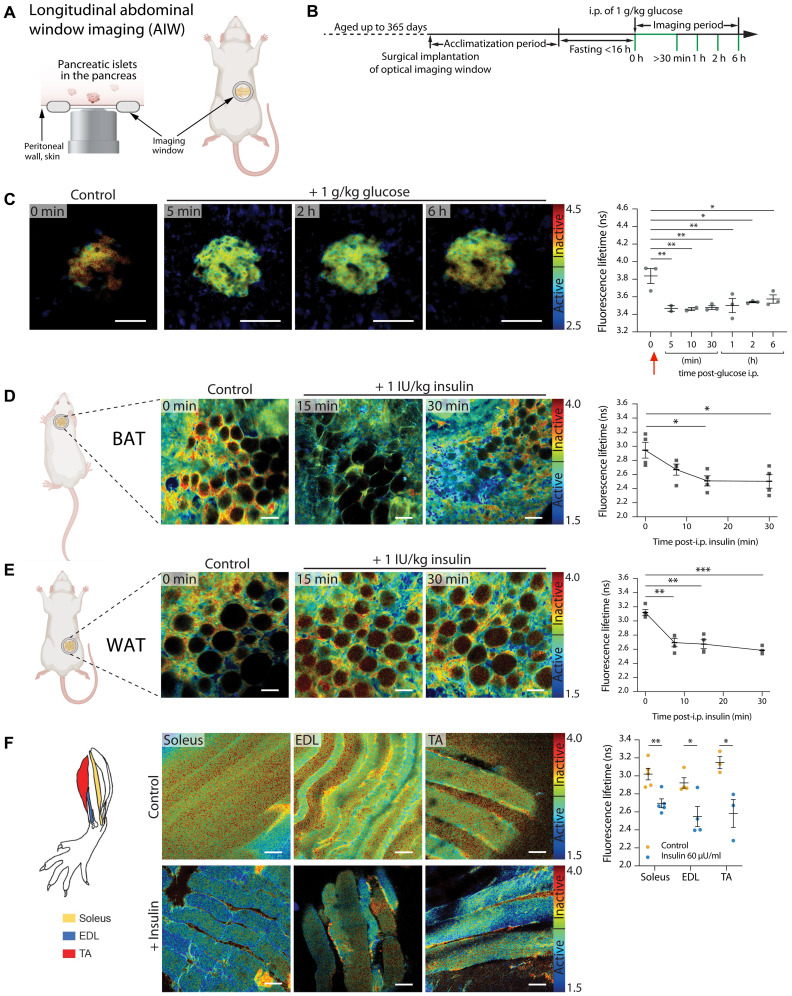
Stimulation of islets of the pancreas with glucose, and of fatty tissues and muscles of the hindleg with insulin, results in spatiotemporal AKT activation in vivo. (**A**) Surgical implantation of optical imaging window over the native pancreas allows for the monitoring of AKT activity in pancreatic islets of *RIP-Cre*;Akt-FRET mice. (**B**) Timeline of implantation of optical imaging window of the primary pancreas and subsequent in vivo imaging of glucose metabolism. (**C**) In vivo imaging of AKT activity in *RIP-Cre*;Akt-FRET mice following bolus administration of glucose (1 g/kg) via intraperitoneal injection showing active AKT after 5 min, 2 hours, and 6 hours. *n* = 2 mice, up to two to three islets imaged per time point, 322 cells quantified in total. Results are means ± SEM. *P* values were determined using an ordinary one-way ANOVA with Dunnett correction for multiple comparisons, and significance is compared to 0-min time point. (**D** and **E**) AKT activation visualized in BAT (D) and WAT (E) following intraperitoneal injection of insulin (1 IU/kg). *n* = 4 mice per tissue per time point, 214 cells quantified in total. Results are means ± SEM. *P* values were determined using a Welch’s *t* test, and significance is compared to 0-min time point. (**F**) AKT activation visualized ex vivo following stimulation with insulin (60 μU/ml) in the soleus, extensor digitorum longus (EDL), and tibialis anterior (TA) muscles. *n* = 3 to 5 mice per tissue. Results are means ± SEM. *P* values were determined using an unpaired *t* test, and significance is compared to untreated controls. Scale bars, 50 μm. ns, *P* > 0.05, *P < 0.05, **P < 0.01, and ***P < 0.001.

In addition to playing a critical role in cancer, AKT signaling has also been demonstrated to be a key signaling node in the glucose metabolism in pancreatic islets (*47*, *48*). Using longitudinal imaging windows, we can further use the Akt-FRET biosensor mouse to explore AKT dynamics in vivo in response to oral gavage of glucose or intraperitoneal injection of insulin. To monitor in vivo AKT signaling, inducible Akt-FRET mice were crossed to a RIP-Cre to allow for pancreatic islet–specific expression of the biosensor. These mice were then engrafted with AIWs (Fig. 6, A and B) (*18*, *19*, *49*) and treated with an intraperitoneal injection of d-glucose. Here, after 5 min post-glucose intraperitoneal AKT was activated and this activation was tracked for up to 6 hours (Fig. 6C), demonstrating the utility of the Akt-FRET biosensor mouse in longitudinal monitoring of metabolic response in individual islets in their native tissue environment.

In response to food ingestion, insulin is released from pancreatic islets to stimulate glucose uptake into insulin-responsive tissues including adipose tissue. This action is primarily mediated by PI3K/AKT signaling pathways. In adipose tissue, the AKT pathway promotes glucose transport and utilization in adipocytes, with this action being mediated via the GLUT4 glucose transporter (*50*). To measure the response of AKT to insulin stimulus, mice were engrafted with optical imaging windows over either their BAT depots, located between the shoulder blades of mice or the subcutaneous WAT in the inguinal region (*51*). Upon insulin challenge, AKT was activated after 15 min in BAT and after 7.5 min in WAT, which was readily assessed for up to 30 min after intraperitoneal injection (Fig. 6, D and E, respectively). We further assessed the response to glucose stimulus directly, which revealed robust AKT activation after 5 min in BAT and 2.5 min in WAT (fig. S5, A and B), uncovering the distinct activation dynamics of AKT in these tissue compartments. This demonstrates the capacity to monitor AKT signaling dynamics faithfully in response to physiologically relevant metabolic stimuli in an intact live setting at distinct sites within the body.

Insulin stimulation to use glucose predominantly occurs in skeletal muscle, with glucose metabolism regulated by the PI3K/AKT signaling pathway in this tissue (*52*). Glucose uptake occurs via AKT-mediated accumulation of GLUT4 at the plasma membrane, leading to subsequent AKT-mediated phosphorylation of GSK3, which metabolizes glucose to glycogen in skeletal muscle cells. We therefore isolated different muscle types with predominantly fast-twitch muscles, such as the tibialis anterior (TA) and the exterior digitorum longus (EDL), and a mixed muscle containing a greater proportion of slow-twitch fibers, such as the soleus (*53*, *54*). In all three muscle types, AKT was readily activated upon insulin stimulation, demonstrating the additional utility of the Akt-FRET biosensor mouse for future assessments of distinct AKT activation dynamics in these functional different tissue types (Fig. 6F). Collectively, these findings reveal how the Akt-FRET biosensor mouse can be used to investigate metabolic changes at a whole-body level.

**Fig. 7.**
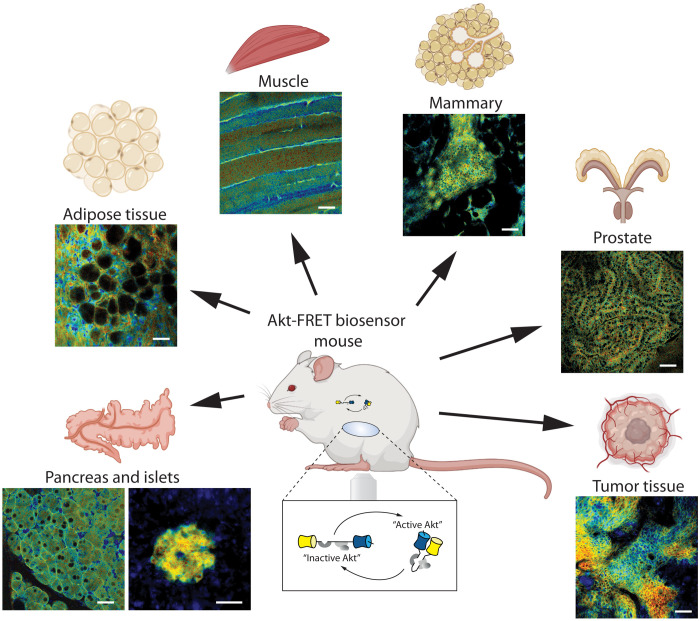
Schematic of spatiotemporal AKT activity mapping in several organ systems in vivo. Optical window–mediated intravital assessment of metabolic and malignancy-mediated AKT signaling in a variety of organs including pancreas, adipose tissue, muscle, mammary, prostate, muscle, and tumor tissue. Scale bars, 50 μm.

In conclusion, we have demonstrated in a large spectrum of organ systems how this biosensor mouse can be used to examine inherent biology and drug targeting in a live real-time setting (Fig. 7). The ability to faithfully monitor and target AKT-PI3K signaling longitudinally in in vivo settings will be paramount for dissecting key stages of malignant and metabolic disease developments. In addition, the spatiotemporally resolved treatment response to specific upstream and downstream inhibitors and how they perform in live target tissue–specific settings will allow for more accurate and molecularly defined treatment approaches in the future.

## DISCUSSION

Intravital imaging allows us to assess cellular behavior in live tissue within an intact cellular ecosystem, thereby facilitating high-fidelity investigations of biology in the tissue or organ of choice (*22*–*24*, *55*). In addition, examining the reversible nature of signaling in response to drug treatment or disease progression at the single-cell level in vivo in this way provides vital information on signaling dynamics in a spatiotemporal manner, which cannot be achieved by conventional biochemical or genomic approaches that often provide single-time point insights into disease states (*22*, *45*). Here, we have shown the utility of the Akt-FRET biosensor mouse in a variety of settings where AKT plays a key role in driving disease such as cancer and where AKT acts as a central signaling node in cellular and tissue homeostasis such as energy metabolism.

We describe the generation and development of the Akt-FRET biosensor mouse that can either ubiquitously express the Akt-FRET biosensor (Akt-FRET ON mice) knocked into the *Rosa26* locus or conditionally induced by tissue-specific Cre recombinases (Akt-FRET OFF mice). Low-level expression from the *Rosa26* locus was chosen to avoid any possible dominant-negative effects of expressing the biosensor in native tissues (see fig. S1, A and B) while simultaneously allowing for a sufficiently bright fluorescent signal for robust intravital FLIM-FRET imaging, as achieved previously (*20*, *24*, *32*, *56*). We used the Akt-FRET biosensor characterized previously (*4*, *5*), which undergoes a conformational change in response to phosphorylation by AKT via a consensus sequence, assuming a FRET conformation and thereby decreasing the lifetime of the donor mTurquoise2 (*57*). Moreover, this fluorophore was chosen as it exhibits a high quantum yield and mono-exponential lifetime decay, allowing us to readily and robustly monitor AKT activity in organ-specific settings (*32*, *58*). Using FLIM allows us to perform intensity-independent assessments of the conformation of the FRET biosensor in 3D in vivo imaging, which is more reliable than ratiometric FRET when imaging in complex nonuniformly light scattering environments such as tissue. FLIM imaging additionally provides us with quantitative readouts of the FRET biosensor activity (*59*, *60*).

The tumor suppressor PTEN regulates AKT activation by hydrolyzing the PI3K product PIP3, which acts upstream of AKT (*61*). PTEN is the second most commonly mutated or lost gene in human cancer (*62*). Here, we demonstrated the utility of the Akt-FRET biosensor mouse in faithfully reporting on dysregulated AKT activity in a variety of cancer types such as PDAC, where heterozygous loss of PTEN in both a KC and KPC setting up-regulated AKT activity and enhanced disease progression (*16*). This was further confirmed in mice with heterozygous loss of PTEN or loss of PTEN lipid phosphatase function (*Pten^G129E^*) mouse models that demonstrated AKT hyperactivation in various tissues (*17*, *61*), and in which through the Akt-FRET biosensor we showed spatial up-regulation of AKT in prostate and breast cancer settings. Here, specifically in prostate cancer lesions, regions of elevated AKT activity could readily be visualized in multiple areas within the primary tumors. This heterogeneous distribution of AKT activity within prostate cancers has been identified by spatial transcriptomics approaches (*63*), although never in such a dynamic, time-resolved manner. In this study, up-regulation of AKT activity was further maintained in organoid culture of prostate cancer tissue, as demonstrated previously in crypt cultures isolated from the Rac1-FRET biosensor mouse (*56*). This illustrates the utility of these biosensor mice in faithfully reporting on signaling cues, including AKT activity, which can be recapitulated in physiologically relevant orthogonal assays such as organoids. Reciprocity between the AR signaling and PI3K/AKT activity has been demonstrated previously (*37*), with AR inhibition causing down-regulation of androgen-dependent FKBP5, which in turn acts as a chaperone for the AKT phosphatase PHLPP, resulting in higher AKT activity (*64*). Here, we treated prostate cancer spheroids with the antiandrogen enzalutamide and saw an up-regulation of AKT activity, as demonstrated previously in PTEN-deficient prostate cancer (*65*). Here, we have, however, shown that this effect may be more evident in cases where there are point mutations in PTEN rather than heterozygous loss. It has been demonstrated previously that these mutations in PTEN down-regulate the function of the second WT copy of PTEN, thus up-regulating PI3K/AKT signaling to a greater extent than heterozygous loss of PTEN (*17*). In addition, point mutations within the catalytic P-loop of PTEN such as *Pten^G129E^*, resulting in loss of PTEN lipid phosphatase function, have been mapped in a variety of cancer settings. Here, we have also shown that inhibition with a proof-of-principle pan-PI3K pathway inhibitor BKM120 can reverse this side effect in antiandrogen-treated conditions. This work points toward the benefit of using more personalized targeted therapy for prostate cancer patients, based on the precise genomic analysis of their tumors, rather than broad, all-comer, one-drug-fits-all treatment approaches. Furthermore, the use of organoid tissue from the Akt-FRET biosensor mouse crossed with precise genetically defined tumor mouse models can be used as an ongoing efficient and preclinical screening tool of specific genetic contexts to mimic genetic aberration and help optimize targeted therapeutic combinations from distinct genetic backgrounds.

Targeting PI3K signaling in PTEN mutated mammary carcinomas has been described previously with effective inhibition of mammary spheroid growth in vitro by the use of BKM120 (*61*). Here, we have shown that this inhibition can be visualized both in the context of 3D invasion of mammary carcinoma cells in 3D organotypic assays and in vivo using optical imaging windows to track the longitudinal targeting efficacy of PI3K inhibition using BKM120 in primary tumors driven by *Pten^G129E^* mutation. This image-guided spatial validation of target inhibition by measuring AKT activity in real time may, in the future, allow for several more specific inhibitors to be explored. Uncovering these spatially and temporally distinct AKT activation dynamics in vivo can additionally guide frequency of treatments that will be required to effectively inhibit AKT in genetically defined personalized disease settings.

Metabolic diseases such as obesity and its severe complication, type 2 diabetes, are associated with insulin resistance where the AKT signaling pathway is impaired, leading to abnormal glucose metabolism in insulin-responsive tissues including BAT, WAT, and skeletal muscles. However, research in the field to date has failed to accurately track AKT activity during this process, due primarily to limitations in the methodological approaches available to study AKT signaling in vivo. The dynamic assessment of AKT signaling response in real time in the native tissue setting has many advantages over conventional approaches. First, it continuously measures intracellular AKT signaling activity upon various interventions in living cells in situ. This is in contrast to Western blotting, which is an end point measurement, providing solely a snapshot view. Second, it does not require the isolation of protein from adipose tissue, thereby having minimal interferences upon protein functions. Easy accessibility of BAT and WAT for imaging purposes provides us with an additional opportunity to monitor dynamics of AKT pathway in adipose tissue to reflect the whole-body metabolic states. While dietary changes from high fat feeding to healthier diets or caloric restriction have proven to be effective to improve insulin resistance in obesity, the timing and duration of these lifestyle changes that produce these metabolic benefits are unclear. In the future, using intravital imaging of the Akt-FRET biosensor mouse could provide a temporal resolution to monitor the changes of metabolic state in BAT and WAT during the transition from a healthy to an obese state following high caloric diet or from an obese to a healthy state following caloric restriction. These changes in AKT activity patterns during these transition periods could enable us to identify critical intervention points for the early and effective treatment of obesity and type 2 diabetes. For example, a recent study suggests that AKT is necessary for optimal BAT functions (*66*). BAT is a specialized organ for adaptive thermogenesis that dissipates energy as heat, and stimulation of BAT thermogenesis and WAT browning are attractive strategies to combat obesity. Therefore, monitoring dynamics of the AKT pathway could provide detailed insights in BAT thermogenesis in response to various interventions or treatments and the Akt-FRET biosensor mouse described here will be a useful tool to monitor therapeutic efficacies of potential anti-obesity medications by monitoring dynamic changes of the AKT pathway within a live physiologically relevant microenvironment.

In conclusion, we have illustrated an array of applications that can be provided by the Akt-FRET biosensor mouse to study longitudinal disease progression and targeting while describing the future utility of this vital resource for a spectrum of disease settings involving upstream or downstream targeting of this critical signaling node in disease etiology.

## MATERIALS AND METHODS

### Experimental design

This study examines the utility of the Akt-FRET biosensor mouse to report on AKT activity in a spatiotemporally resolved manner using FLIM-FRET of the donor mTurquoise2 as a primary readout. This is assessed in tissue homeostasis, malignancy and metabolic contexts, and associated organ systems on a single-cell level. In vitro organotypic invasion assays, 3D spheroid cultures, and ex vivo culturing of pancreatic islets was done in a minimum of three independent biological replicates with three technical replicates per treatment group unless otherwise indicated in the figure legends. Mouse numbers used in in vivo experiments are outlined in the corresponding figure legends.

FLIM-FRET analysis of the Akt-FRET biosensor was conducted on a minimum of 30 cells per group in a minimum of three independent biological repeats unless otherwise indicated in the figure legends for in vitro and in vivo experiments. Unless otherwise indicated, all immunofluorescence (IF) and IHC analyses were performed on six representative regions of interest (ROIs) in organotypic matrices and five ROIs in IF experiments. Study endpoints for in vivo experiments were in compliance with Garvan/St Vincent’s Animal Ethics Committee (16/13, 19/13, and 22/10) and the Australian code of practice for care and use of animals for scientific purposes.

### Statistical analysis

Statistical analysis was performed using GraphPad Prism (GraphPad Software Inc., CA) with statistical significance given as ns (not significant), *P* > 0.05, *P < 0.05, **P < 0.01, ***P < 0.001, and ****P < 0.0001. Data were analyzed by unpaired Welch’s *t* test, one-way analysis of variance (ANOVA), or two-way ANOVAs with the corresponding corrections as indicated in the respective figure legends.

### Animals

Animal experiments were conducted in accordance with the Garvan/St Vincent’s Animal Ethics Committee guidelines (16/13, 19/13, and 22/10) and in compliance with the Australian code of practice for care and use of animals for scientific purposes. Mice of both genders and mixed backgrounds were kept in individual ventilated, isolated cages on a 12-hour light/12-hour dark cycle and fed ab libitum. The conditional Rosa26 locus Akt-FRET biosensor mice were generated as previously described (*31*). Briefly, G4 BL/6-129 hybrid embryonic stem (ES) cells that harbor an FlpE recombinase compatible docking cassette at the endogenous Rosa26 locus were used to target the incoming conditional Akt-FRET-pA cassette. Correctly targeted parental NeoR ES cells were used in CD1 diploid embryo-G4 ES cell aggregation experiments to generate strong (90 to 100%) ES cell–derived chimeras as judged by brown coat color. Germline transmission of the conditional Akt-FRET allele was confirmed by polymerase chain reaction (PCR). Akt-FRET mice were crossed to a CMV-Cre to remove the LSL site and allow for ubiquitous expression of the biosensor (Akt-FRET ON mice). LSL-Akt-FRET mice were further crossed to specific Cre driver lines, such as Pdx1-Cre, Rip-Cre, and NPY-Cre. *KRas^G12D/+^;Pdx1-Cre* (KC) or *KRas^G12D/+^;p53^R172H/+^;Pdx1-Cre* (KPC) models were further crossed to the LSL-Akt-FRET biosensor mouse as well as a conditional *Pten^floxed/+^* model to assess Akt signaling in the context of pancreatic cancer. Last, Akt-FRET ON mice were crossed to whole-body *Pten^+/−^* and *Pten^G129E/+^* models described previously (*17*).

### Cell culture

Parental G4 BL/6-129 hybrid ES cells were maintained under standard ES cell culture conditions with media supplemented with leukemia inhibitory factor (LIF). In vitro Cre-excised daughter cells that have the potential to express the Akt-FRET biosensor were generated by electroporation of correctly targeted conditional parental Rosa26-LSL-Akt-FRET-pA ES cells with pCAGGS-Cre-IRES-puro cassette. Puro^R^ ES cell clones were picked, expanded, and confirmed to have deleted the floxed STOP cassette by PCR (*31*). These Cre-excised Akt-FRET ON ES cells were used in subsequent analysis.

*Pten^G129E/+^* mammary carcinoma cells were maintained in Dulbecco’s modified Eagle’s medium (DMEM) (GIBCO; containing 25 mM glucose, 4 mM l-glutamine, and 1 mM sodium pyruvate) supplemented with 10% fetal bovine serum (FBS), 1% penicillin/streptomycin (P/S), insulin (5 mg/ml), EGF (10 ng/ml), and cholera toxin A (10 ng/ml) and telomerase-immortalized fibroblasts (TIFs) in DMEM supplemented with 10% FBS and 1% P/S at 37°C and 5% CO_2_. Cell lines were checked and confirmed free of mycoplasma.

Mouse embryonic fibroblasts (MEFs) were split into 10-cm dishes and fluorodishes and grown to confluence, before treatment with mitomycin C for 3 hours. Onto these feeder plates, ESC clones 5 and 6 were thawed, grown to a high density, and treated ± EGF (20 ng/ml) for 30 min.

### Prostate organoid culture

3D prostate organoid cultures were adapted from a protocol described previously (*41*). Briefly, the urogenital system of aged-matched WT, *Pten^+/−^*, and *Pten^G129E/+^* male mice was isolated and seminal vesicles, the vas deferens, bladder, and urethra were carefully removed from the prostate lobes. Minced prostate lobes were digested in collagenase/hyaluronidase (5 mg/ml; STEMCELL Technologies) shaking for 1.5 hours at 37°C, washed with adDMEM/F12+/+/+ (containing P/S, 10 mM Hepes, and 2 mM GlutaMAX), and centrifuged for 5 min at 150*g* at 4°C. The pellet was resuspended in 1 ml of TrypLE and allowed to digest for another 15 min at 37°C. Cells were counted and, after subsequent centrifugation in 10 ml of adDMEM/F12+/+/+, resuspended in Matrigel to plate 10,000 cells per 10 μl of Matrigel droplet per well in a black flat glass bottom 96-well plate. After solidification of the Matrigel at 37°C, the growth medium adDMEM/F12+/+/+ [containing 1× B27, 1.25 mM *N*-acetylcysteine, EGF (50 ng/ml), Noggin (100 ng/ml), murine R-spondin (500 ng/ml), 200 nM A83-01, and 10 μM Y-27632 dihydrochloride] was added and organoids were allowed to form for a period up to 14 days. The growth factor–containing medium was refreshed every 3 to 4 days, and organoids were passaged one time at day 7. Organoids were treated at days 9 and 10 with either 1 μM BKM120, 100 nM enzalutamide alone, or in combination and imaged before and after treatment to quantify Akt activity.

### Organotypic invasion

3D organotypic invasion assays were set up as described previously (*21*, *44*, *67*). Briefly, collagen was isolated from 12 to 14 frozen adolescent rat tails, and the tendons were dissolved in precooled 0.5 M acetic acid at 4°C for 48 to 72 hours. The remaining sheaths were removed by filtering the collagen extract through a mesh towel before the addition of 10% (w/v) NaCl to precipitate the collagen. After stirring for 1 hour, the extract was centrifuged for 30 min at 10,000 rpm at 4°C. The precipitate was then redissolved in 400 ml of 0.25 M precooled acetic acid at 4°C for 24 hours. Subsequent dialysis was performed against six to eight changes of 5 liters of Millipore water containing 17.5 mM acetic acid. The dialyzed collagen was centrifuged at 14,000 rpm for 1.5 hours, and the supernatant was placed in a sterile flask and stored at 4°C. For approximately 12 organotypic matrices, 25 ml of collagen was mixed with 3 ml of MEM 10x media (GIBCO) and neutralized by titration of 0.22 M NaOH. FBS (3 ml) was used to resuspend TIFs (*68*) and added to the collagen mixture. For each matrix, 2.5 ml of this collagen/fibroblast mix per matrix was then plated in six-well culture dishes and allowed to solidify for 30 min at 37°C and 5% CO_2_. Two milliliters of DMEM growth medium (containing 10% FBS and 1% P/S) was added to each well, and the matrices were detached to contract freely for approximately 10 to 14 days at 37°C and 5% CO_2_. The medium was changed every 2 to 3 days. The organotypic matrices were cleared of the fibroblasts by the addition of puromycin (10 mg/ml) to the medium for 3 days and subsequent washing of the matrices with phosphate-buffered saline three times. *Pten^G129E/+^* mammary carcinoma cells were seeded onto the matrices in a 24-well plate at 10^5^ cells/ml overnight. Tripods were created from sterile stainless steel grids and placed in 6-cm dishes. Growth medium was added, and the matrices were placed on the grids from an air/liquid interface. A triplicate of matrices per condition was allowed to invade in DMEM, 10% FBS, and 1% P/S supplemented with insulin (5 mg/ml), EGF (10 ng/ml), and cholera toxin A (10 ng/ml) for up to 14 days and treated during the last 4 days of invasion with vehicle, 1 μM BKM120, or 50 μM rapamycin, respectively. Following this, the matrices were imaged using the FLIM-FRET analysis outlined below and fixed in 10% neutral-buffered formalin. They were then processed for histological analysis, by paraffin embedding, microtomy, and hematoxylin and eosin (H&E), and automated pNDRG1, pS6, and Ki-67 staining were performed as described below.

### Immunohistochemistry

Organotypic matrices and tissues were fixed in 10% neutral-buffered formalin for 24 hours and stored in 70% ethanol until processing. Paraffin-embedded tissues were cut to 4-μm sections using Leica Microtome RM2235, and sections were placed on Superfrost Plus Slides. Slides were deparaffinized with xylene, rehydrated with washes in decreasing concentrations of ethanol, and performed on Leica Autostainer XL. H&E staining and counterstaining with hematoxylin (Shandon Instant Haematoxylin Kit) were further performed on Leica Autostainer XL. The Aperio Scanscope image capture device was used to acquire images of all slides at ×20 magnification. Stainings for pNDRG1 and Ki67 were performed as described previously (*5*) with a Leica Bond RX fully automated research stainer and a Leica Bond Polymer Refine Detection kit. Heat-induced epitope retrieval at 93°C with Bond Epitope Retrieval Solution 2 (EDTA-based, pH 9) was performed for 20 to 30 min for pNDRG1, Ki67, pS6, and GFP stainings. The slides were stained using either a 1:500 dilution of the Ki67 rabbit antibody (RM-9106-S1, Thermo Fisher Scientific), a 1:1000 dilution of the pNDRG1 (Thr^346^) antibody (5482, Cell Signaling Technology), a 1:600 dilution of the pS6 (Ser^240/244^) antibody (35708, Cell Signaling Technology), or 1:250 for GFP antibody (A-11122, Thermo Fisher Scientific). Slides were counterstained, coverslipped, and scanned as described above. Ki67-, pS6-, and pNDRG1-stained sections were quantified using QuPath (v0.2.0-m9) (*69*), with 3,3'-diaminobezidine (DAB) and hematoxylin optical densities computed for each pixel using color deconvolution. A watershed cell detection based on the hematoxylin counterstain was used to identify nuclei within these regions. Single cells were identified by applying a 5-μm nuclei detection radius and the average DAB optical density computed for each cell. Using a constant threshold applied, cells were classed as either positive or negative for DAB staining and values were averaged for a minimum of three regions of interest per organotypic matrix per condition.

### Western blotting

Tissue lysates were prepared in radioimmunoprecipitation assay lysis buffer [50 mM Hepes, 1% (v/v) Triton X-100, 0.5% sodium deoxycholate, 0.1% SDS, 0.5 mM EDTA, 50 mM NaF, 10 mM NA_3_VO_4_, a 1× protease inhibitor cocktail (cOmplete Mini, Roche), and 1× phosphatate inhibitor PhosSTOP tablet (Roche)]. Protein concentration was determined by Bradford (Bio-Rad) assay, and volumes were adjusted according to measurements. Gel electrophoresis was performed using 4 to 12% Bis-Tris Protein Gels (NuPage, Thermo Fisher Scientific), and proteins were transferred onto polyvinylidene difluoride membranes (Immobilon-P, Millipore). Membranes were subsequently blocked at room temperature in 5% Tris-buffered saline with Tween® 20 (TBST) skim milk in TBST. Primary antibodies were diluted in 1% bovine serum albumin in TBST at 1:1000 for anti-GFP (A-11122, Thermo Fisher Scientific), 1:1000 for total AKT (9272, Cell Signaling Technology), 1:1000 for pAKT (Thr^308^) (4056, Cell Signaling Technology), 1:1000 for pAKT (Ser^473^) (4060, Cell Signaling Technology), and 1:1000 for β-actin (A2228, Sigma-Aldrich) and incubated overnight at 4°C. After TBST was washed, the membranes were incubated with horseradish peroxidase–linked secondary anti-rabbit and anti-mouse antibody (GE Healthcare, 1:5000, diluted in 1% skim milk/TBST) for 1 hour at room temperature. Enhanced chemiluminescence (ECL) and Ultra-ECL reagents (Western Lighting Plug-ECL, PerkinElmer) were used to visualize the bioluminescent signal imaged on a Fusion FX (Vilber) system. β-Actin was used as a loading control, and the levels of total and phospho-AKT in three Akt-FRET ON mice were compared to the organs of interest of three WT mice.

### Optical imaging windows

Mammary and abdominal imaging windows for intravital imaging were surgically engrafted in mice described previously (*20*, *21*). All surgical instruments were autoclaved by steam sterilization or by using a bead sterilizer (Germinator), and equipment and surfaces were sterilized by wiping down with F10 disinfectant followed by 70% ethanol. Twenty-four hours before the surgeries, cyanoacrylate was applied to the edges of a titanium ring (Russel Symes & Company) and a coverslip of 12 mm diameter was placed into the inset of that ring and kept in 70% ethanol until implantation. Twenty-four hours before and at least 72 hours after surgery, carprofen (Rimadyl; 5 mg/kg) was administered to the mice in the drinking water. Anesthesia was induced and maintained by inhalation of the gas isoflurane using a calibrated vaporizer. To prevent eye dehydration, the eyes were lubricated with eye ointment (LacriLube). Anesthesia with regular reflex testing on the footpad was maintained throughout the entire procedure. Subcutaneous injection of 100 ml of buprenorphine (0.075 mg/kg) was used for further pain management before and 6 hours after the surgery. The incision site was shaved to clear any hair, depilated using hair removal cream (Nair), and disinfected using 0.5% chlorhexidine/70% ethanol. For MIWs, an incision was made in the skin overlying the palpable tumor or subcutaneous adipose tissue in the inguinal region for WAT and between the shoulder blades for BAT deposits. A purse string suture was then applied through the skin. For AIWs, an additional incision was performed through the peritoneal wall and the purse string suture was placed, through both the skin and muscle wall as previously described (*18*). The imaging window was then placed into the incision by careful insertion of the skin into the lateral groove of the window, and the sutures were tied off. Any trapped air beneath the window was aspirated. The mice were allowed to recover for 72 hours after surgery and weaned off carprofen before in vivo imaging was performed. To minimize damage to the window by the mice and cage surroundings, metal food hoppers and plastic domes are removed from the cages and feed supplied in food trays on the floor of the cage. Paper-mache domes with the bottom of the entry hole removed are supplied as cage enrichment along with tissues as nesting material. To aid recovery, mice were supplied with recovery gel and/or sunflower seeds.

### In vivo and ex vivo imaging, single-cell FLIM analysis, and motion correction

Excised organs were imaged ex vivo for a maximum of 30 to 60 min after removal. Mice bearing an optical window were imaged under 1 to 2% isoflurane on a heated stage (Digital Pixel, UK) before and after respective gavage or intraperitoneal injection of BKM120 (30 mg/kg), glucose (1 g/kg), or insulin (1 U/kg). In vivo imaging was performed as described previously on a Leica DMI 6000 SP8 inverted confocal microscope using a 25 × 0.95 numerical aperture (NA) water immersion objective. The Ti:sapphire femtosecond laser (Coherent Chameleon Ultra II, Coherent) excitation source operating at 80 MHz was tuned to a pumping wavelength of 840 nm. RLD-HyD detectors were used with 435/40-nm and 483/40-nm bandpass emission filters to detect the second-harmonic generation (SHG) of collagen I and mTurquoise2, respectively. Images were acquired at a line rate of 700 Hz, 512 × 512 pixel, and at a total of 203 frames per image. Realignment of the data was performed using Galene (v2.0.2) (*46*) using the warp realignment mode, 10 realignment points, a smoothing radius of 2px, and a realignment threshold of 0.4 applied for the SHG channel and 0.6 for the mTurquoise2 signal. Single-cell analysis was performed using FLIMfit (v5.1.1) (*70*) by drawing ROIs encompassing the cell cytoplasm and thresholding. For the prostate, mammary, adrenal tumor, and lymphomas, a cutoff value of 3.40 ns was chosen to classify cells as active or inactive for Akt. Changes in fluorescent lifetime on a single-cell level in three to four regions per mouse per condition were plotted and analyzed using Prism 8 (GraphPad).
