# Supplementary material for: Monitoring AKT activity and targeting in live tissue and disease contexts using a real-time Akt-FRET biosensor mouse
Source: Sci Adv. 2023 Apr 26;9(17):eadf9063. doi: 10.1126/sciadv.adf9063 (PMC10132756; doi:10.1126/sciadv.adf9063)
Supplement: Supplementary file 1 — Figs. S1 to S5 Legends for movies S1 and S2 [file sciadv.adf9063_sm.pdf]

Science Advances
AAAS

Supplementary Materials for

Monitoring AKT activity and targeting in live tissue and disease contexts using a real-time Akt-FRET biosensor mouse

James R. W. Conway *et al.*

Corresponding author: Paul Timpson, p.timpson@garvan.org.au; Max Nobis, max.nobis@vib.be; Jody J. Haigh, jody.haigh@umanitoba.ca

Sci. Adv. **9**, eadf9063 (2023)
DOI: 10.1126/sciadv.adf9063

The PDF file includes:

Figs. S1 to S5
Legends for movies S1 and S2

Other Supplementary Material for this manuscript includes the following:

Movies S1 and S2

List of acronyms

3D	Three Dimensional
AIW	Abdominal Imaging Window
AR	Androgen Receptor
BAT	Brown Adipose Tissue
CMV	Cytomegalovirus
EDL	Exterior Digitorum Longus
EGF	Epidermal Growth Factor
Enz	Enzalutamide
EPCAM	Epithelial Cellular Adhesion Molecule
ESC	Embryonic Stem Cell
FKBP5	FK506 Binding Protein 5
FLIM	Fluorescence Lifetime Imaging Microscopy
FOV	Field Of View
FOXO	Forkhead Box O
FRET	Förster Resonance Energy Transfer
FRT	Flippase Recognition Target
GEMM	Genetically Engineered Mouse Models
GFP	Green Fluorescent Protein
GLUT	Glucose Transporter Family
GPCR	G-Protein-Coupled Receptors
GSK3	Glycogen Synthase Kinase 3
IHC	Immunohistochemistry
KC	LSL-KRas$^{G12D/+}$;Pdx1-Cre
KPC	LSL-KRas$^{G12D/+}$;LSL-p53$^{R172H/+}$;Pdx1-Cre

LSL	Lox-Stop-Lox
MIW	Mammary Imaging Window
mTORC1	Mechanistic Target of Rapamycin Complex 1
mT2	mTurquoise2
NDRG1	N-Myc Downstream Regulated 1
PanIN	Pancreatic Intraepithelial Neoplasm
PBD	Phosphate Binding Domain
PDAC	Pancreatic Ductal Adenocarcinoma
PH	Pleckstrin Homology domain
PHLPP	PH domain Leucine-rich repeat-containing Protein Phosphatase
PI3K	Phosphoinositide 3-kinase
PIK3CA	Phosphatidylinositol-4,5-Biphophate 3-Kinase Catalytic Subunit Alpha
PIP2	Phosphatidylinositol-4,5-bisphosphate
PIP3	Phosphatidylinositol-3,4,5-triphosphate
PKA	Protein Kinase A
PKB	Protein Kinase B (Akt)
PKC	Protein Kinase C
PTEN	Phosphatase and Tensin Homolog
RMCE	Recombinase-Mediated Cassette-Exchange
RTK	Receptor Tyrosine Kinase
SEM	Standard Error of the Mean
SHG	Second Harmonic Generation
TA	Tibialis Anterior
WAT	White Adipose Tissue
WT	Wild Type

YFP	Yellow Fluorescent Protein
YPet	YFP for Energy Transfer

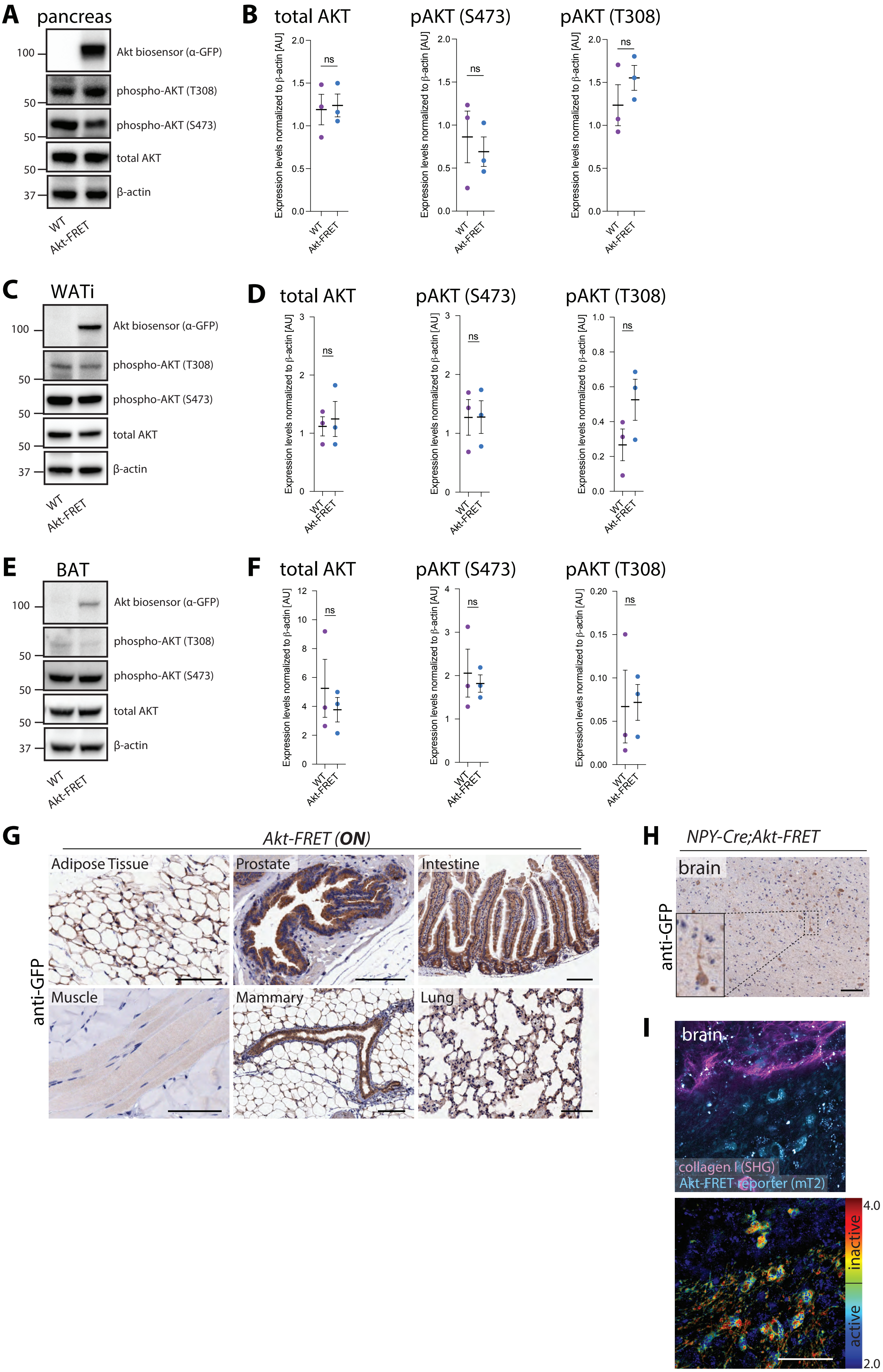

A pancreas
100
50
50
50
37
Akt biosensor (α-GFP)
phospho-AKT (T308)
phospho-AKT (S473)
total AKT
β-actin
WT
Akt-FRET
B total AKT
pAKT (S473)
pAKT (T308)
Expression levels normalized to β-actin [AU]
ns
C WATi
D total AKT
E BAT
F total AKT
G Akt-FRET (ON)
anti-GFP
Adipose Tissue
Prostate
Intestine
Muscle
Mammary
Lung
H NPY-Cre;Akt-FRET
brain
I
collagen I (SHG)
Akt-FRET reporter (mT2)
4.0
inactive
active
2.0

Fig S1: Validation of tissue specific expression of the Akt-FRET biosensor by Western blot and Immunohistochemistry and *NPY-Cre* specific driven expression and AKT activity monitoring.

A. Expression of Akt-FRET biosensor in the pancreas of WT and Akt-FRET 'ON' mice as detected by immunoblot of total and phospho-AKT levels (Ser473 and Thr308) quantified between WT and Akt-FRET mice in **B**. n = 3 mice per group. Results; mean ± SEM. p-values were determined using Welch's *t*-test. ns $p > 0.05$. **C**,**D** Expression of Akt-FRET biosensor in the inguinal white adipose tissue (WATi) of WT and Akt-FRET 'ON' mice as detected by immunoblot of total and phospho-AKT levels (Ser473 and Thr308) quantified between WT and Akt-FRET mice. n = 3 mice per group. Results; mean ± SEM. p-values were determined using Welch's *t*-test. ns $p > 0.05$. **E**,**F** Expression of Akt-FRET biosensor in the brown adipose tissue (BAT) of WT and Akt-FRET 'ON' mice as detected by immunoblot of total and phospho-AKT levels (Ser473 and Thr308) quantified between WT and Akt-FRET mice. n = 3 mice per group. Results; mean ± SEM. p-values were determined using Welch's *t*-test. ns $p>0.05$. **G.** Akt-FRET biosensor expression by anti-GFP IHC in various organs of the Akt-FRET 'ON' biosensor mouse, including adipose tissue, muscle, prostate, mammary tissue, intestine and lungs. Scale bars: 100 µm. **H.** Neuropeptide Y (NPY) tissue compartment specific expression of the Akt-FRET biosensor in *NPY-Cre* crossed mice in brain neurons as detected by anti-GFP IHC. Scale bar: 100 µm. **I.** *Ex vivo* imaging performed on an inverted 2-photon microscope of AKT activity in the arcuate nucleus of the hypothalamus by *NPY-Cre* driven Akt-FRET biosensor (mTurquoise2/mT2, cyan) and collagen visualized by second harmonic generation (SHG, magenta) with corresponding representative intensity-merged maps of mTurquoise2 fluorescence lifetimes. Scale bars: 50 µm. ns $p > 0.05$.

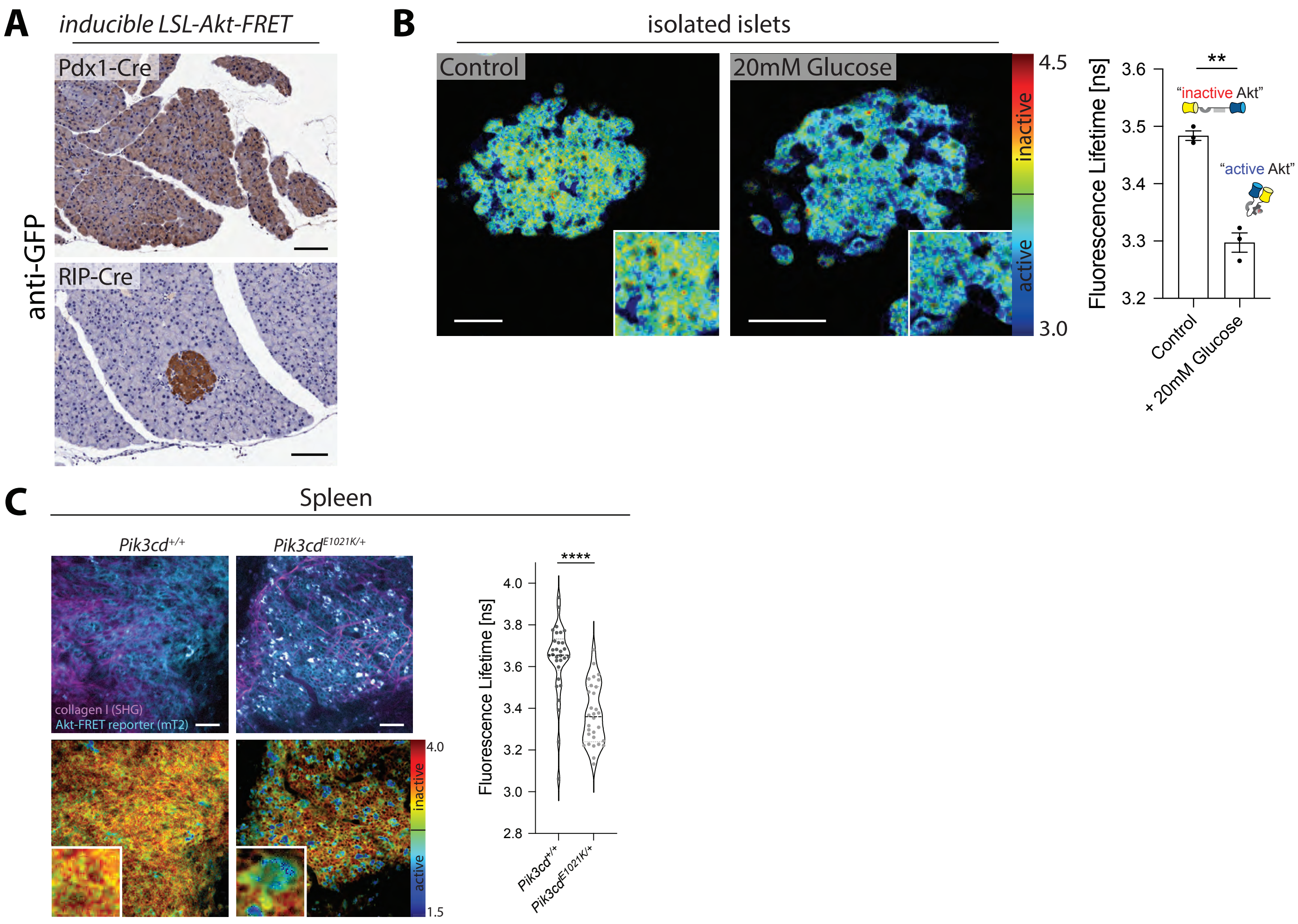
A
inducible LSL-Akt-FRET
anti-GFP
Pdx1-Cre
RIP-Cre
B
isolated islets
Control
20mM Glucose
4.5
3.0
inactive
active
Fluorescence Lifetime [ns]
3.6
3.5
3.4
3.3
3.2
**
"inactive Akt"
"active Akt"
Control
+ 20mM Glucose
C
Spleen
Pik3cd+/+
Pik3cdE1021K/+
collagen I (SHG)
Akt-FRET reporter (mT2)
4.0
1.5
****
Fluorescence Lifetime [ns]
4.0
3.8
3.6
3.4
3.2
3.0
2.8

Fig S2: *Ex vivo* stimulation of pancreatic islets isolated from pancreata with glucose results in spatiotemporal AKT activation and *Pik3cd*$^{E1021K/+}$ driven hyperactivation of AKT in leukocytes of the spleen *in vivo*.

A. Anti-GFP immunohistochemistry (IHC) of the Akt-FRET biosensor in inducible Akt-FRET mice crossed to pancreas specific *Pdx1-Cre* and pancreatic islets specific *RIP-Cre* drivers. Scale bars: 100 µm. **B.** AKT activity in isolated pancreatic islets stimulated with 20mM glucose. n = 3 mice, 253 cells, Results; mean ± SEM. p-value was determined using Welch's *t*-test, significance is compared to untreated control colonies. **C.** Increasing AKT activity in the spleen of *Pik3cd*$^{E1021K/+}$ mice as imaged through optical windows *in vivo*. n = 60 cells quantified. Results; mean ± SEM. p-value was determined using Welch's *t*-test, significance is compared to untreated control colonies. Scale bars, 50 µm. **p<0.01, **** p < 0.0001.

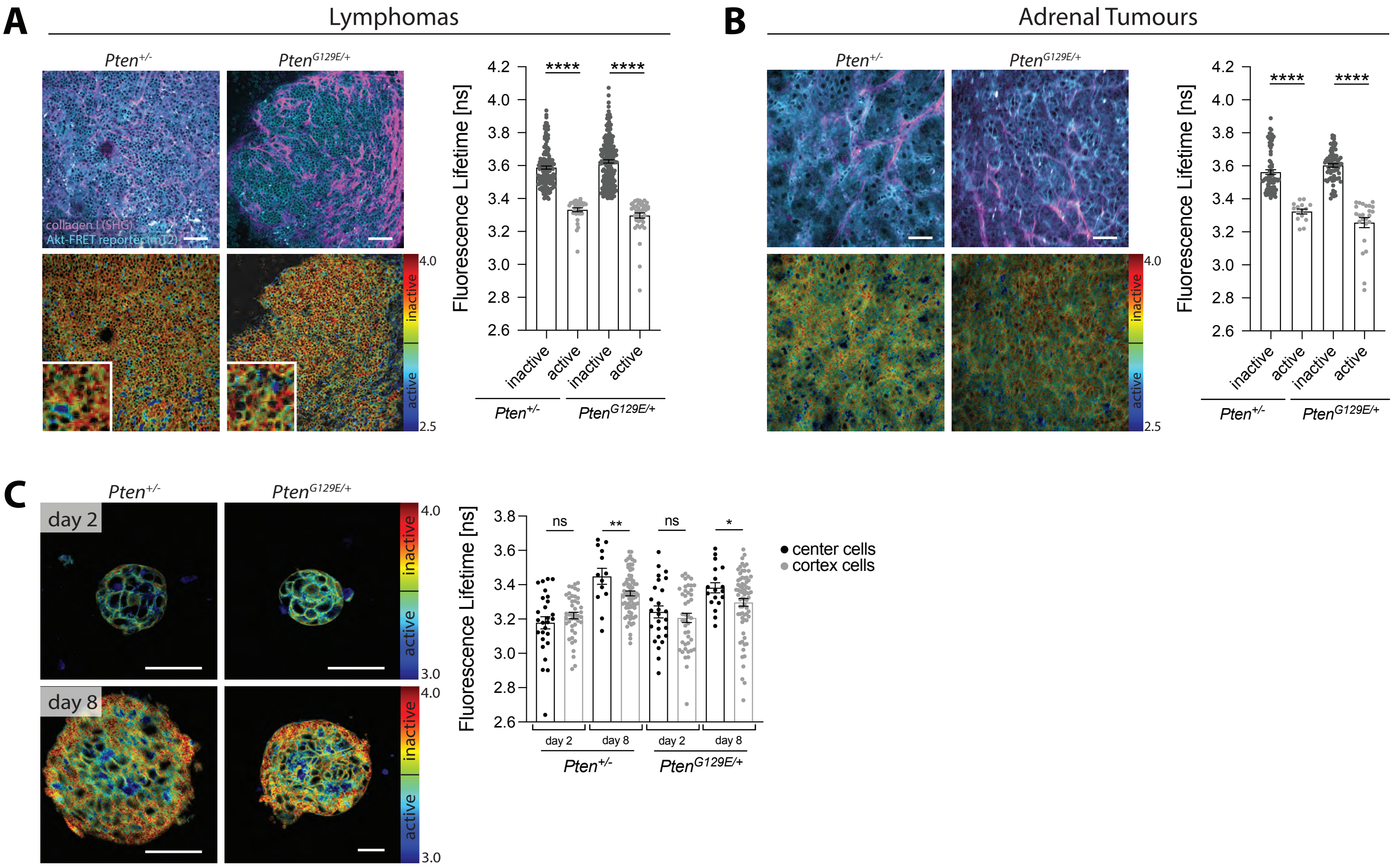

A
Lymphomas
Pten+/-
PtenG129E/+
collagen I (SHG)
Akt-FRET reporter (m12)
4.0
inactive
active
2.5
Fluorescence Lifetime [ns]
****
****
inactive
active
inactive
active
Pten+/-
PtenG129E/+
B
Adrenal Tumours
Pten+/-
PtenG129E/+
4.0
inactive
active
2.5
Fluorescence Lifetime [ns]
****
****
inactive
active
inactive
active
Pten+/-
PtenG129E/+
C
Pten+/-
PtenG129E/+
day 2
day 8
4.0
inactive
active
3.0
Fluorescence Lifetime [ns]
ns
**
ns
*
day 2
day 8
day 2
day 8
Pten+/-
PtenG129E/+
center cells
cortex cells

Fig S3: Elevated AKT signaling in *Pten*$^{+/-}$ and *Pten*$^{G129E/+}$ mutant lymphomas and adrenal gland tumors and spatial distribution of AKT activity in prostate cancer spheroids.

A,B. Increasing AKT activity in locally restricted zones of lymphomas (**A**) and adrenal tumors (**B**) following heterozygous loss of PTEN (*Pten*$^{+/-}$) and expression of mutant PTEN (*Pten*$^{G129E/+}$). n = 4-17 mice per genotype, 625 cells in total analyzed. Results; mean ± SEM. p-values were determined using Brown-Forsythe and Welch ANOVA with Dunnett correction for multiple comparisons, significance is compared to inactive cells. **C.** Isolated prostate spheroids cultured for up to 14 days with representative images on day 2 and 8 showing spatially defined increased AKT activity in the centers of both *Pten*$^{+/-}$ and *Pten*$^{G129E/+}$ spheroids cultured for 8 days. n = 3 spheroids per condition per timepoint, 321 cells in total (data from Figure 3D). Results; mean ± SEM. p-values were determined using Welch's *t*-test, significance is compared to the "center" of each condition. Scale bars, 50 µm. ns p > 0.05, * p < 0.05, ** p < 0.01, **** p<0.0001.

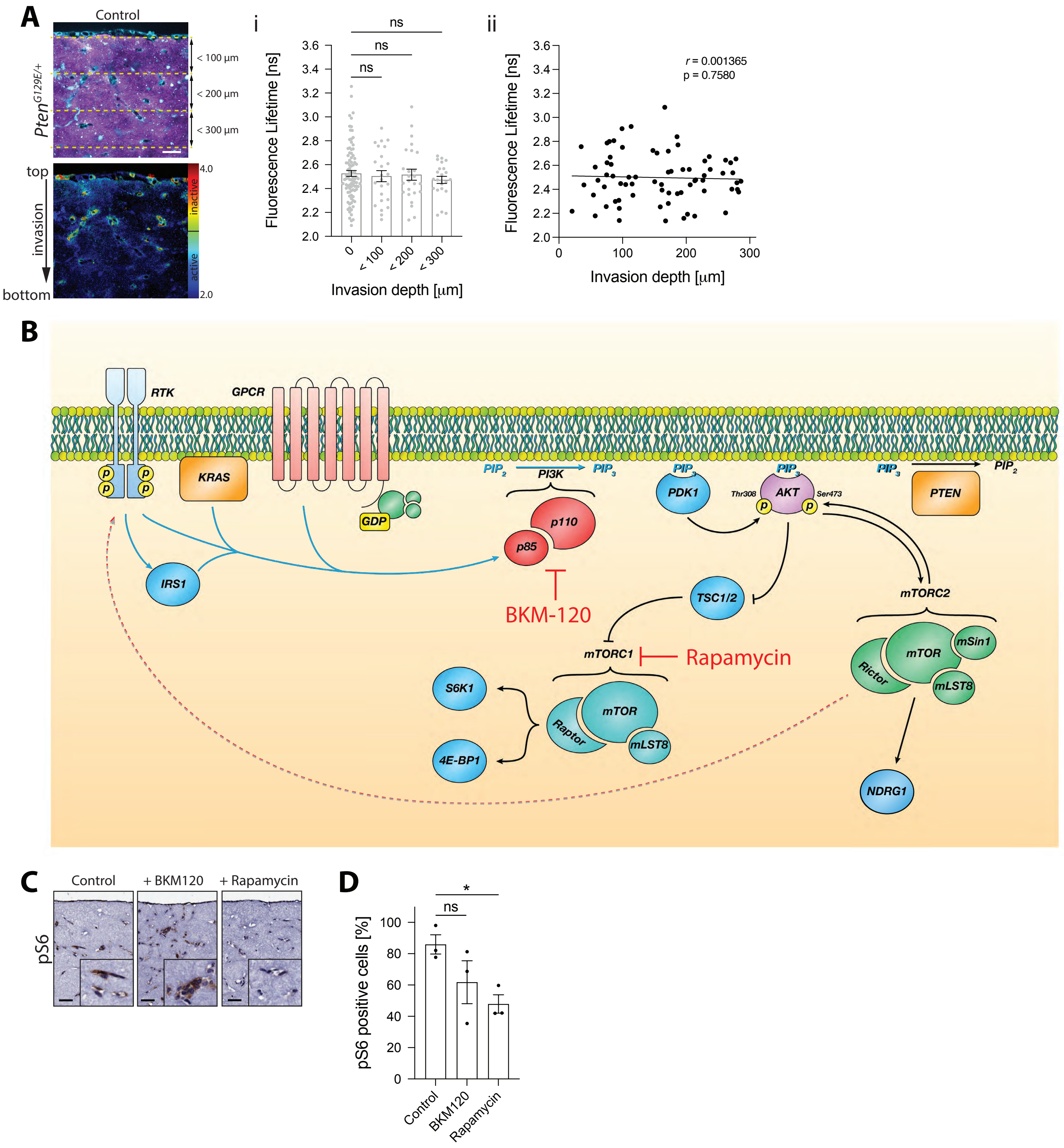
A
Control
Pten G129E/+
< 100 µm
< 200 µm
< 300 µm
top
invasion
bottom
4.0
inactive
active
2.0
i
ns
ns
ns
Fluorescence Lifetime [ns]
3.6
3.4
3.2
3.0
2.8
2.6
2.4
2.2
2.0
0
< 100
< 200
< 300
Invasion depth [µm]
ii
r = 0.001365
p = 0.7580
Fluorescence Lifetime [ns]
0
100
200
300
Invasion depth [µm]
B
RTK
GPCR
KRAS
GDP
IRS1
PIP2
PI3K
PIP3
p110
p85
BKM-120
PDK1
Thr308
AKT
Ser473
PTEN
TSC1/2
mTORC1
Rapamycin
S6K1
4E-BP1
mTOR
Raptor
mLST8
mTORC2
mSin1
Rictor
NDRG1
C
Control
+ BKM120
+ Rapamycin
pS6
D
*
ns
pS6 positive cells [%]
100
80
60
40
20
0
Control
BKM120
Rapamycin

Fig S4: Correlation of invasion depth to AKT activity, schematic of PI3K pathway inhibition and pS6 reduction upon Rapamycin treatment in invading *Pten*$^{G129E/+}$ driven mammary tumor cells.

A. Quantification of the AKT activity in mutant *Pten*$^{G129E/+}$ cells invading into organotypic matrices at different depths 0µm,< 100µm, < 200µm and < 300µm respectively (i) and correlation analysis of AKT activity with invasion depth on a single cell level (ii). n = 3, with a total of 165 cells quantified (data from Figure 4C). Results; mean ± SEM. p-values were determined using ordinary one-way ANOVA and Pearson correlation coefficient analysis. Scale bar: 50 µm. **B.** Schematic of PI3K pathway inhibition, upstream of AKT with the PI3K inhibitor BKM120 and downstream of AKT by the mTORC1 inhibitor Rapamycin. **C**. Representative images of phospho-S6 (pS6) staining performed on *Pten*$^{G129E/+}$ cell line invading on organotypic matrices +/- BKM120, Rapamycin quantified in **D.** n = 3 per cell line. Scale bars: 100 µm. Results; mean ± SEM. p-values were determined using ordinary one-way ANOVA, significance is compared to Ctr. ns p>0.05, *p<0.05.

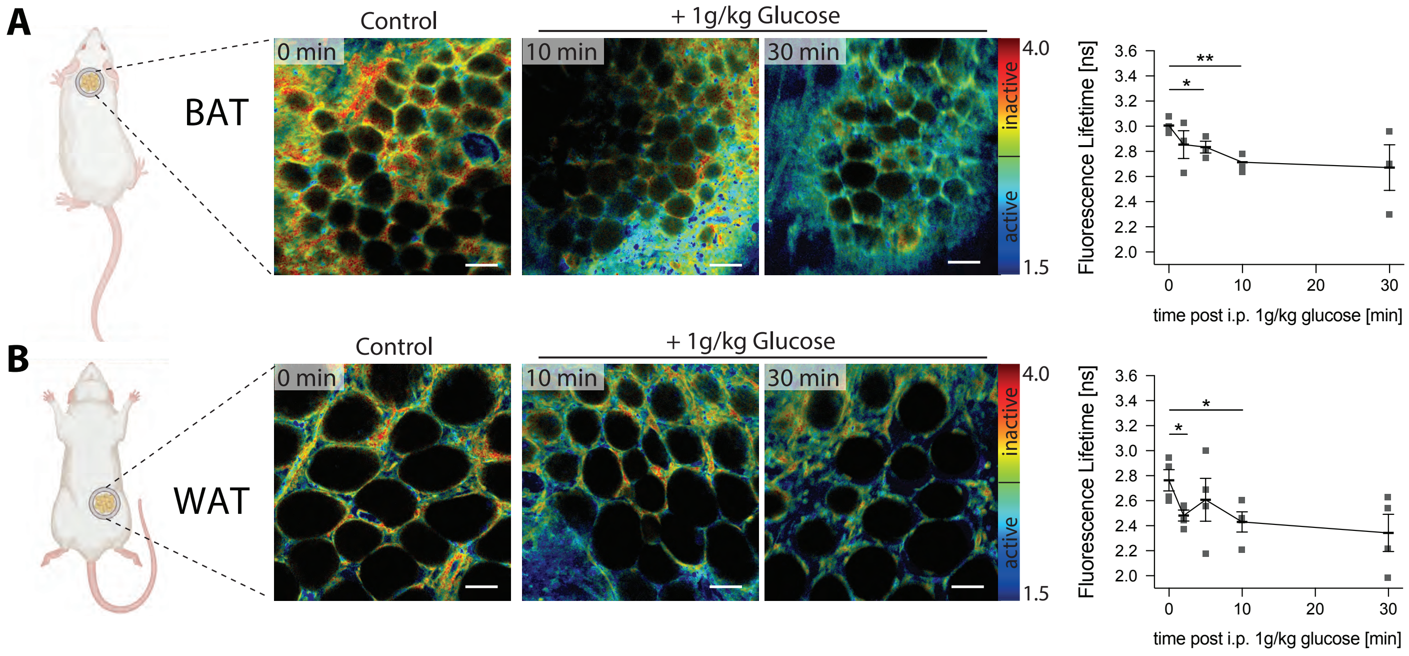

A
Control
+ 1g/kg Glucose
BAT
0 min
10 min
30 min
4.0
inactive
active
1.5
Fluorescence Lifetime [ns]
time post i.p. 1g/kg glucose [min]
B
Control
+ 1g/kg Glucose
WAT
0 min
10 min
30 min
4.0
inactive
active
1.5
Fluorescence Lifetime [ns]
time post i.p. 1g/kg glucose [min]

Fig S5: *In vivo* stimulation of brown and white fatty tissue with glucose results in spatiotemporal AKT activation.

A,B. AKT activation visualized in brown adipose tissue (BAT, **A**) and white inguinal adipose tissue (WAT, **B**) following i.p. injection of 1g/kg glucose. n = 3-4 mice per tissue per timepoint. Results; mean ± SEM. p-values were determined using a Welch's *t*-test, significance is compared to 0 minutes timepoint. ns p>0.05, *p<0.05, **p<0.01.

Other Supplementary Material for this manuscript includes

Movie S1. Expression of the Akt-FRET biosensor in a variety of tissues including pancreas, brown adipose tissue (BAT), white adipose tissue (WAT), skeletal muscle, liver, lung, prostate and mammary gland. Z-stack images of the intensity of the Akt-FRET biosensor (mTurquoise2/mT2, cyan) and collagen visualized by second harmonic generation (SHG, magenta) are displayed as well as FLIM heatmap showing spatially resolved AKT activity (scale bar, 50 µm).

Movie S2. Galene-mediated correction of motion artefacts introduced by respiration and heartbeat during intravital imaging of a *RIP-Cre*;Akt-FRET islet in the pancreas using abdominal optical imaging windows (scale bar, 50 µm).
